# Timing matters: real-world effectiveness of early combination of biologic and conventional synthetic disease-modifying antirheumatic drugs for treating newly diagnosed polyarticular course juvenile idiopathic arthritis

**DOI:** 10.1136/rmdopen-2019-001091

**Published:** 2020-02-02

**Authors:** Bin Huang, Tingting Qiu, Chen Chen, Yin Zhang, Michael Seid, Dan Lovell, Hermine I Brunner, Esi M Morgan, Patient Centered Adaptive Treatment Strategies (PCATS), Michelle Adams, Timothy Beukelman, Anne Kocsis, Melanie Kohlheim, Jeff Guo, Stephanie Gray, Jinzhong Liu, Alivia Neace, Siva Sivaganisan, Stacey Woeste, Xiaomeng Yue, Janet Zahner

**Affiliations:** 1 Division of Biostatistics and Epidemiology, Cincinnati Children’s Hospital Medical Center, Cincinnati, Ohio, USA; 2 Department of Pediatrics, Cincinnati Children's Hospital Medical Center, Cincinnati, Ohio, USA; 3 Division of Pulmonary Medicine, Cincinnati Children’s Hospital Medical Center, Cincinnati, Ohio, USA; 4 Division of Rheumatology, Cincinnati Children's Hospital Medical Center, Cincinnati, Ohio, USA

**Keywords:** juvenile idiopathic arthritis, DMARDs (biologic), DMARDs (synthetic), outcomes research, disease activity

## Abstract

**Objectives:**

To compare real-world effectiveness of two adaptive treatment strategies of disease-modifying antirheumatic drugs (DMARDs) in treating children with newly diagnosed polyarticular course juvenile idiopathic arthritis (pcJIA): early aggressive use of biologic DMARDs (bDMARDs) in combination with conventional synthetic DMARDs (csDMARDs) versus conservative delayed use of bDMARDs following the initial csDMARD prescription.

**Methods:**

A single-centre newly diagnosed DMARD-naive pcJIA patient database (n=465) was derived from the electronic medical records between 1 January 2009 and 31 December 2018. The primary study endpoints were clinical Juvenile Arthritis Disease Activity Score (cJADAS) at 6 and 12 months following the first DMARD prescription. The secondary study endpoint was Pediatric Quality of Life Inventory (PedsQL) generic total score at 12 months. Averaged causal treatment effects were assessed using a Bayesian non-parametric casual inference method.

**Results:**

Both cJADAS and PedsQL improve over time, regardless of the treatment strategies. Compared with the conservative approach, early aggressive approach is more effective in reducing cJADAS (mean −2.17, 95% CI −3.77 to −0.56) by 6 months. Adding bDMARD after 6 months to the initial treatment provides very little added benefit. The averaged treatment effect was 6.35 (95% CI −5.89 to 18.58) improvement in PedsQL at 12 months.

**Conclusions:**

Timing matters—early aggressive use with bDMARDs is more effective than conservative delayed treatment in lowering disease activity after 6 and 12 months of treatment.

**Key messages**

**What is already known about this subject?**

- Recent treatment guidelines recommended adaptive treatment strategies admitting biologic disease-modifying antirheumatic drugs (bDMARDs) at different timing, adaptive to patient’s response. Previous trials suggested early aggressive combination of conventional synthetic DMARDS (csDMARDS)+bDMARDs is more effective than csDMARDs only.

**What does this study add?**

- This comparative effectiveness research study compared the early combination cs+bDMARD versus delayed use of bDMARD in treating children with newly diagnosed polyarticular course juvenile idiopathic arthritis (pcJIA). The results suggested that early use of bDMARD can effectively reduce disease activity by 6 months of treatment. Adding bDMARD at 6 months provides very little benefit for the 12-month outcome.
- The study is novel in the study design and analytical methods. It took new patient DMARD-naive study design. It applied a novel Bayesian non-parametric causal inference method. Electronic medical records are used to offer real-world evidence particularly for evaluating effectiveness of adaptive treatment strategies.

**How might this impact on clinical practice?**

- This study suggests timing matters. Early use of bDMARDs is more effective than delayed bDMARD use in achieving earlier and sustained improvement in treating children with newly diagnosed pcJIA.

## Introduction

Juvenile idiopathic arthritis (JIA) is the most common rheumatological disease in children and a cause of childhood disability. The global prevalence of JIA is approximately 19.4 per 100 000 for girls and 11.0 per 100 000 for boys.^1^ The cause of childhood arthritis is unknown, and the current understanding of the disease aetiology and pathogenesis are limited.^2^ Achieving inactive disease earlier was found to be associated with less joint damage and functional impairment.^3 4^ The advent of disease-modifying antirheumatic drugs (DMARDs), particularly biologic DMARDs (bDMARDs), in the past two decades have revolutionised the treatment approaches to JIA, making it possible to target for inactive disease as the treatment goal. Despite the advanced DMARD treatment, still about 50% of the patients with JIA failed to achieve inactive disease during long-term follow-up,^5^ and most of them had detectable joint damage.^6^ Recent treatment guidelines recommend adaptive treatment strategies (ATSs), such as the consensus treatment plans^7^ and treat-to-target strategies^8^ for JIA. Similar ATS are recommended for adults with rheumatoid arthritis.^9–11^ These ATSs adjust treatment based on patient’s disease activity and response to the previous treatments.^12^

Currently, the conventional treatment practice is to treat patients on the conventional synthetic DMARDs (csDMARDs) first and only introduce bDMARDs if poor prognoses are present. Alternatively, a more aggressive approach is to take the early combination of csDMARDs and bDMARDs (cs+bDMARDs) approach first, then tapering off or stop a medication after the disease activity is brought under control. Evidence from randomised control trials (RCTs) suggests early aggressive use of bDMARDs in combination with methotrexate works better than methotrexate alone in achieving early clinical responses.^13^ However, real-world evidence of clinical effectiveness is lacking.^7 9^

This study aims to evaluate real-world evidence on the effectiveness of early aggressive use of cs+bDMARD versus the conservative strategy of using bDMARD later following the initial prescription(s) in treating children with newly diagnosed polyarticular course JIA (pcJIA).

## Methods

### Study design and patient population

Data were extracted from the electronic medical record (EMR) system for 2082 patients with JIA seen at a large US Midwest paediatric rheumatology clinic from 1 January 2009 to 31 December 2018. Eligible patients were 1–19 years old, DMARD-naive, and newly diagnosed pcJIA including subtypes of polyarthritis, oligoarthritis, psoriatic arthritis, enteritis-related arthritis and undifferentiated arthritis according to the International League of Associations for Rheumatology.^14^ Patient must be diagnosed with pcJIA in at least two distinct visits by paediatric rheumatologists and had a rheumatology clinic visit within 6 months after the diagnosis. Patients with the comorbid conditions of inflammatory bowel disease, coeliac disease and trisomy 21 were excluded.^7^

### Treatment

**Figure 1:**
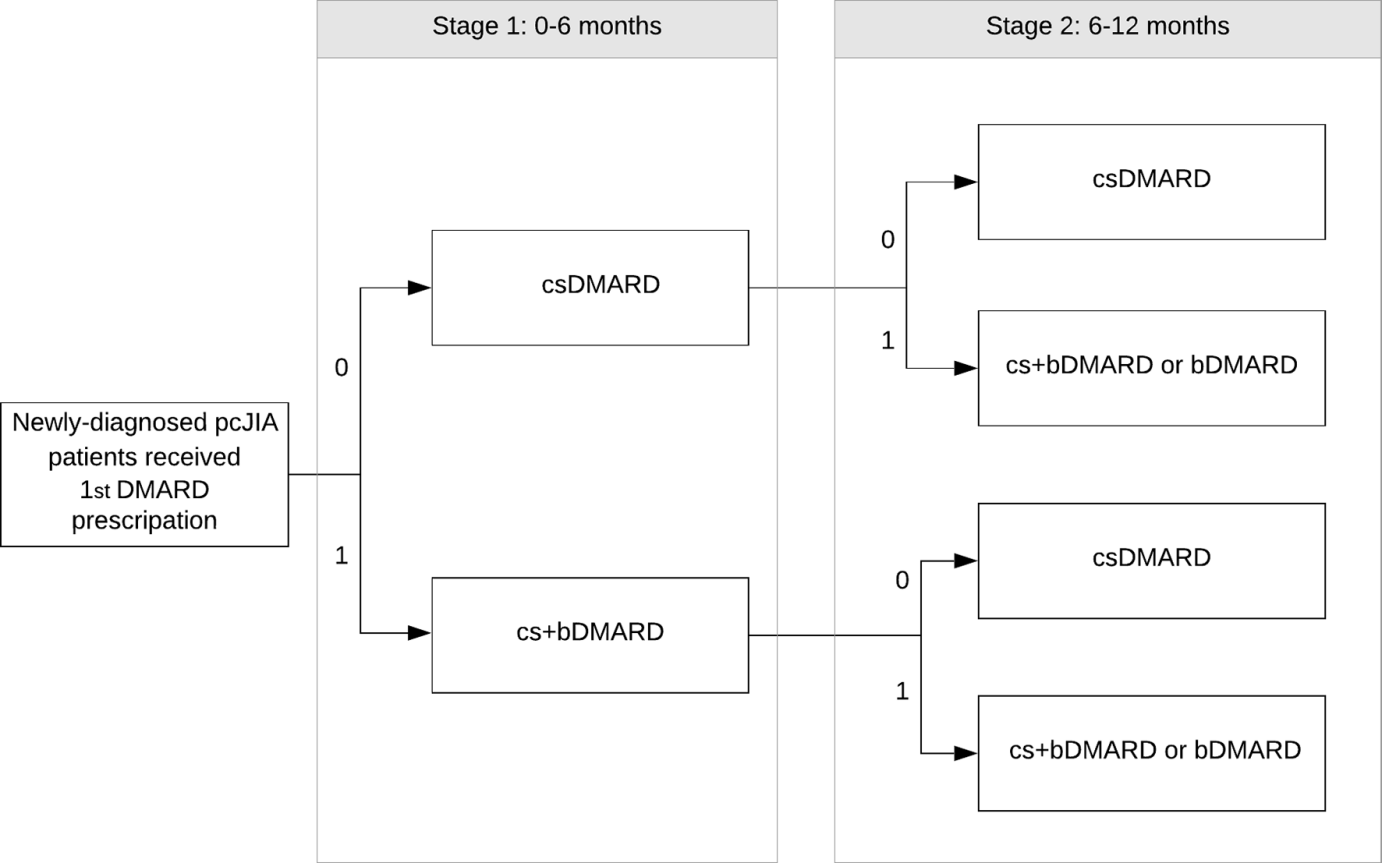
Adaptive treatment strategies for disease-modifying antirheumatic drug prescriptions at stage 1 and stage 2. 0 indicates conservative treatment approach and 1 indicates aggressive treatment approach. DMARD, disease-modifying antirheumatic drug; bDMARD, biologic disease-modifying antirheumatic drug; csDMARD, conventional synthetic disease-modifying antirheumatic drug; cs+bDMARD, combination of conventional synthetic and biologic disease-modifying antirheumatic drug; pcJIA, polyarticular course juvenile idiopathic arthritis.

The adaptive treatments (figure 1) were determined based on the concurrent medication prescriptions recorded in the EMR at all clinical encounters. Patients assigned to early aggressive treatment strategy group were those received both bDMARD and csDMARD prescriptions within 2 months. Patients on the conservative comparator group were those initiated on csDMARDs and did not receive any bDMARD in at least 3 months. The time when patients received their first DMARD prescription was the baseline (0 month). The follow-up visits at 3, 6 and 12 months were identified based on patients’ subsequent clinical visits and changes in DMARD prescriptions. Any changes in medication prescriptions were recorded and compared with the previous prescription. Based on the prescription changes, patients were further allocated into the second-stage adaptive groups based on initiation or dropping of bDMARDs by 6 months of follow-up (figure 1).

### Outcomes

The primary outcomes were the clinical Juvenile Arthritis Disease Activity Score (cJADAS) at the 6-month and 12-month follow-up visits. The cJADAS10 ranges 0–30, summarising the physician global assessment of disease activity (range, 0–10), patient/parent global assessment of well-being (range, 0–10) and active joint count truncated at 10.^15^ Higher cJADAS indicates higher disease activity. The cJADAS was calculated for all visits at 0, 6 and 12 months using observations from clinical encounters that fall within the 1-month time window. If more than one clinical encounter occurred within the window, then an averaged value of the specific core measures was used.

The secondary outcome was health-related quality of life assessed by the Pediatric Quality of Life Inventory (PedsQL) generic module. The PedsQL generic total score ranges from 0 to 100, with a higher score indicating a better quality of life.^16^ Since patients were only asked to fill out the PedsQL questionnaire on an annual basis, the observed score was assigned to the nearest visit date for each patient within a 3-month window. Patients who had less than 12 months of follow-up were excluded when analysing the PedsQL outcome at 12 months.

### Covariates

Demographic variables included age, race, gender and insurance type. Disease characteristics included JIA subtype, age of diagnosis, year of diagnosis, disease duration at the baseline and age at the initiation of DMARDs. Biological variables included rheumatoid factor, antinuclear antibodies and erythrocyte sedimentation rate. Other than the three core measures used in the calculation of cJADAS, patient-reported pain, duration of morning stiffness and physician assessment of total number of joints with limited range of motion were also collected. All these covariates were considered in the statistics causal inference analyses in order to correct for the confounding-by-indication bias.

Over the course of the treatment, the clinical measures such as the biological variables, patient-reported data (pain and stiffness), cJADAS components and active joint counts may change over time. These measures, along with the duration of follow-up, were considered as time-varying covariates in the analyses.

### Patient and public involvement

This study partners with the Paediatric Rheumatology Care and Outcomes Improvement Network to share study results with healthcare professionals and parents of patients with JIA. This study also invited two parents of patients with JIA to serve in the stakeholder advisory panel to comment on study design.

### Statistical analyses

At the baseline, the patient demographic, insurance and disease characteristics were compared between the two groups using χ^2^ test or t-test. Using clinical observational data, unlike the RCTs, treatments were assigned deliberately by patients’ disease status. Therefore, sicker patients tend to receive more aggressive treatment. Bayesian causal inference with Gaussian process (GP) prior has been used to address such confounding-by-indication bias.^17–19^ The GPMatch uses GP covariance function as a matching tool similarly as the Mahalanobis distance matching method.^19^ Balance plots present the averaged mean and the mean absolute dispersion difference between any given patient and his or her matched neighbour on all baseline covariates after GPMatch adjustment. Technical details of the GPMatch method are presented in the Methodology online supplementary appendix.

10.1136/rmdopen-2019-001091.supp1 Supplementary data

Missing baseline data were imputed by applying Bayesian multivariate missing data imputation using the hierarchically coupled mixture model with local dependence method.^20^ Sensitivity analyses evaluated the sensitivity of the study results to potential existence of unmeasured confounders (eg, health-related quality of life).

## Results

### Patient characteristics

**Figure 2:**
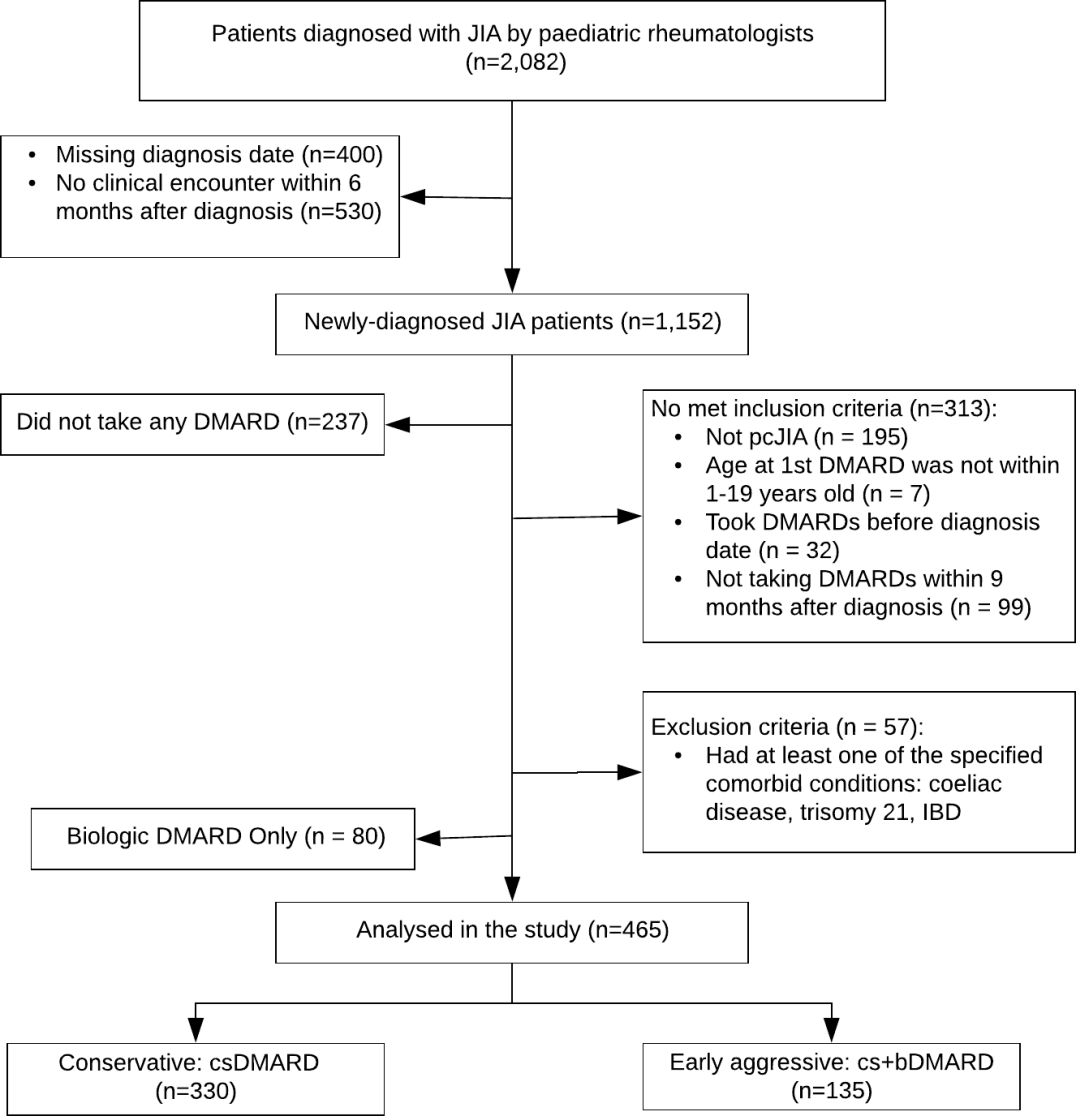
Flow chart of study eligibility screening. JIA, juvenile idiopathic arthritis; pcJIA, polyarticular course juvenile idiopathic arthritis; DMARD, disease-modifying antirheumatic drug; bDMARD, biologic disease-modifying antirheumatic drug; csDMARD, conventional synthetic disease-modifying antirheumatic drug; cs+bDMARD, combination of conventional synthetic and biologic disease-modifying antirheumatic drug; IBD, inflammatory bowel disease\.

Out of 545 eligible patients, 330 were treated on conservative strategy, and 135 were treated in early aggressive strategy. Since we only compared early aggressive versus conservative approach, patients with bDMARD only (n=80) were not included in this study. Detailed patient eligibility screening is summarised in figure 2.

Table 1 presents the baseline characteristics of patients by treatment group. The average time from date of diagnosis to baseline visit were 0.10 year (SD 0.16), similar in both groups (p=0.36). Confounding by indication was clearly evident. Patients on early aggressive treatment had significantly more active disease at baseline (eg, mean±SD of cJADAS 16.08±7.14 vs 12.39±5.91; p<0.0001). The mean and SD of follow-up duration were 0.50±0.06 year and 1.00±0.06 year at 6 and 12 months.

**Table 1:** Patient characteristics at the time of initiation of the first DMARD treatment

Baseline variable	Conservative: csDMARD (n=330)		Early aggressive: cs+bDMARD (n=135)		
	N	Mean±SD	N	Mean±SD	P value*
Age (years)	330	9.81±5.08	135	10.24±4.74	0.40
Age of diagnosis (years)	330	9.70±5.11	135	10.14±4.78	0.39
Year of diagnosis	330	2013.57±3.14	135	2013.12±2.88	0.15
Onset age (years)	265	8.18±4.86	110	8.77±5.04	0.29
Disease duration at diagnosis (years)	266	1.40±2.24	109	1.66±2.97	0.35
Time since diagnosis (years)	330	0.11±0.16	135	0.09±0.16	0.36
cJADAS10 (0–30)	203	12.39±5.91	92	16.08±7.14	<0.0001
Active joint count (0–71)	296	7.53±8.87	127	11.97±11.77	<0.0001
Patient/parent global assessment of well-being (0–10)	294	3.39±2.46	122	4.54±2.69	<0.0001
Physician global assessment (0–10)	221	4.14±2.45	98	5.08±2.68	0.002
Limited range of motion (0–71)	296	5.51±7.27	127	9.33±11.30	<0.0001
Erythrocyte sedimentation rate (mm/h)	175	19.79±19.54	85	32.33±29.39	<0.0001
Global pain NRS (0–10)	298	4.15±2.71	124	5.12±2.65	0.0008
PedsQL generic total	165	67.58±17.53	67	61.35±19.24	0.018

bDMARD, biologic disease-modifying antirheumatic drug; cJADAS, clinical Juvenile Arthritis Disease Activity Score; csDMARD, conventional synthetic disease-modifying antirheumatic drug; NRS, Numerical Rating Scale; PedsQL, Pediatric Quality of Life Inventory.

**Table:** 

	N	%	N	%	P value
Female	236	71.5	98	72.6	0.81
Race					0.81
White or Caucasian	293	88.8	117	86.7	
Black or African American	21	6.4	9	6.7	
Other	12	3.6	7	5.2	
Unknown	4	1.2	2	1.5	
JIA subtype					0.03
Poly RF−	118	35.8	61	45.2	
Poly RF+	26	7.9	15	11.1	
Oligo	105	31.8	26	19.3	
Other	81	24.5	33	24.4	
Insurance					0.83
Public	82	24.8	33	24.4	
Private	222	67.3	89	65.9	
Other	26	7.9	13	9.6	
Morning stiffness					0.002
None	65	19.7	16	11.9	
15 min	35	10.6	12	8.9	
>15 min	116	35.2	73	54.1	
Unknown	114	34.5	34	25.2	
Uveitis ever					0.96
No	143	43.3	57	42.2	
Yes	10	3.0	4	3.0	
Unknown	177	53.6	74	54.8	
Elevated C reactive protein	43	13.0	38	28.1	<0.0001
Rheumatoid factor—positive	18	5.5	18	13.3	0.004
Antinuclear antibodies—positive	33	10.0	21	15.6	0.09
HLA-B27—present	13	3.9	10	7.4	0.12
Previous treatment with NSAID	252	76.4	79	58.5	0.0001
Previous treatment with prednisone	20	6.1	13	9.6	0.17

*P values of χ^2^ for categorical variables and Student t-test for continuous variables.

HLA-B27, Human leukocyte antigen B27; JIA, juvenile idiopathic arthritis; NSAID, non-steroidal anti-inflammatory drug; RF, rheumatoid factor.

### Treatment patterns

Of the 330 patients initiated on csDMARD, the majority (n=319, 96.67%) were prescribed methotrexate. Fifteen patients had less than 3 months of follow-up, and an additional 10 patients had less than 6 months of follow-up. At the 3-month follow-up, of the 315 patients followed up, 285 (90.5%) patients remained on the same DMARDs and 24 (7.6%) stopped DMARDs. One patient switched from methotrexate to sulfasalazine. At the 6-month follow-up, of the 300 patients followed up, 133 (44%) started on bDMARD, 149 (50%) stayed on the same initial prescription and 18 (6%) patients were off DMARDs.

Of the 135 patients who received early combination prescription, 81 (60%) were on methotrexate and etanercept, and 36 (26.7%) were on the methotrexate and adalimumab combination. All 135 patients had a 3-month follow-up visit; majority (112, 85.5%) of them stayed on the same prescription. At the 6-month visit, of 129 patients followed up, 109 (84.5%) continued on the same prescription and 20 (15.5%) discontinued from bDMARD.

### Primary outcome: cJADAS at the 6-month and 12-months follow-up

**Figure 3:**
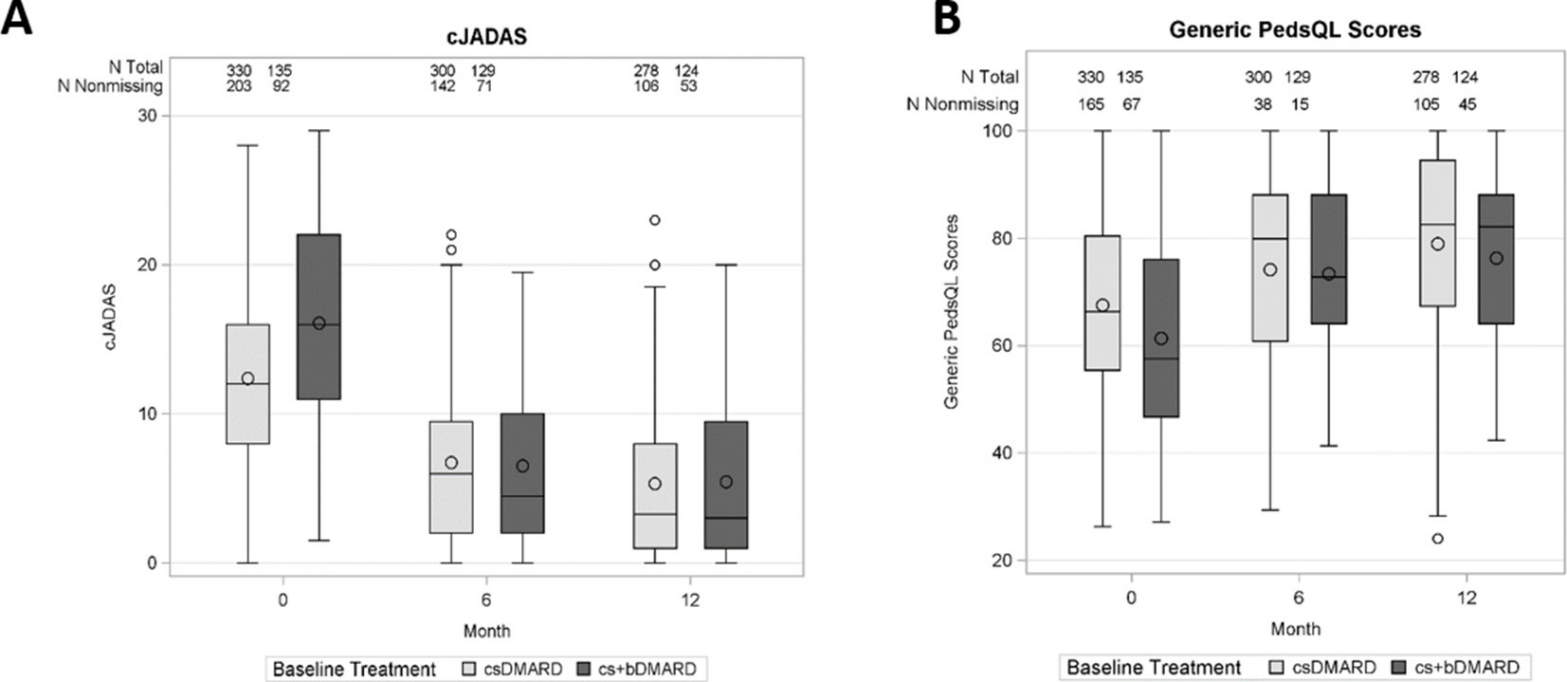
Box plots for cJADAS and PedsQL scores by treatment group at baseline, 6 months and 12 months. cJADAS, clinical Juvenile Arthritis Disease Activity Score; csDMARD, conventional synthetic disease-modifying antirheumatic drug; cs+bDMARD, combination of conventional synthetic and biologic disease-modifying antirheumatic drug; PedsQL, Pediatric Quality of Life Inventory.

Although patients on the early aggressive combination presented higher disease activities at the baseline, the two groups were no longer statistically different at the follow-up visits. Respectively, the cJADAS (mean±SD) changed from 16.08±7.14 and 12.39±5.91 at the baseline, to 6.47±5.68 and 6.91±5.68 at 6-month follow-up, and 5.45±5.64 and 5.25±5.32 at the 12-month follow-up. The box–whisker plots of cJADAS over time grouped by initial prescription are presented in figure 3A.

**Figure 4:**
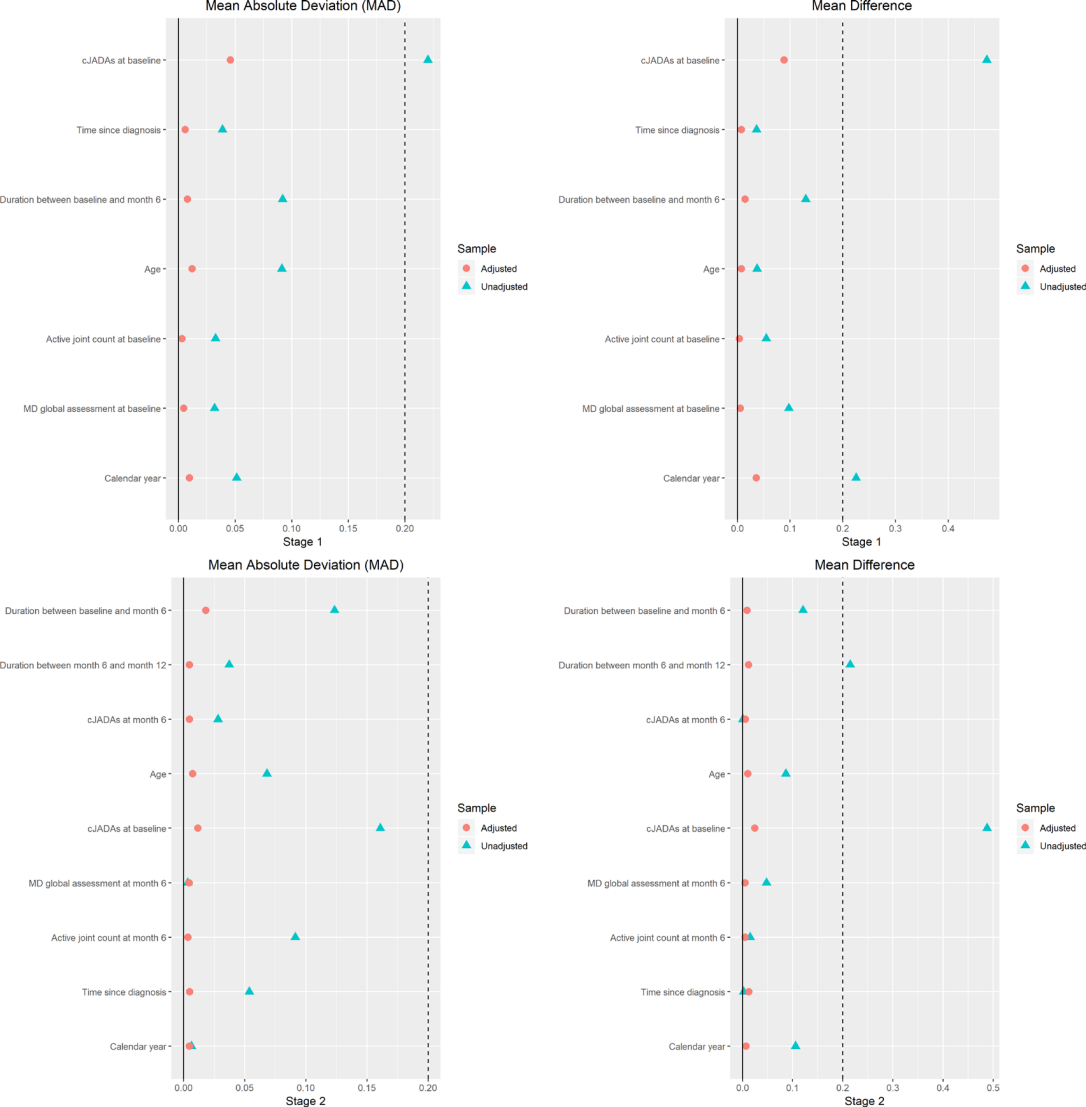
Balance check for covariates used in the causal inference model, reporting in mean absolute deviation (MAD) and mean difference. cJADAS, clinical Juvenile Arthritis Disease Activity Score; MD, Doctor of Medicine.

**Figure 5:**
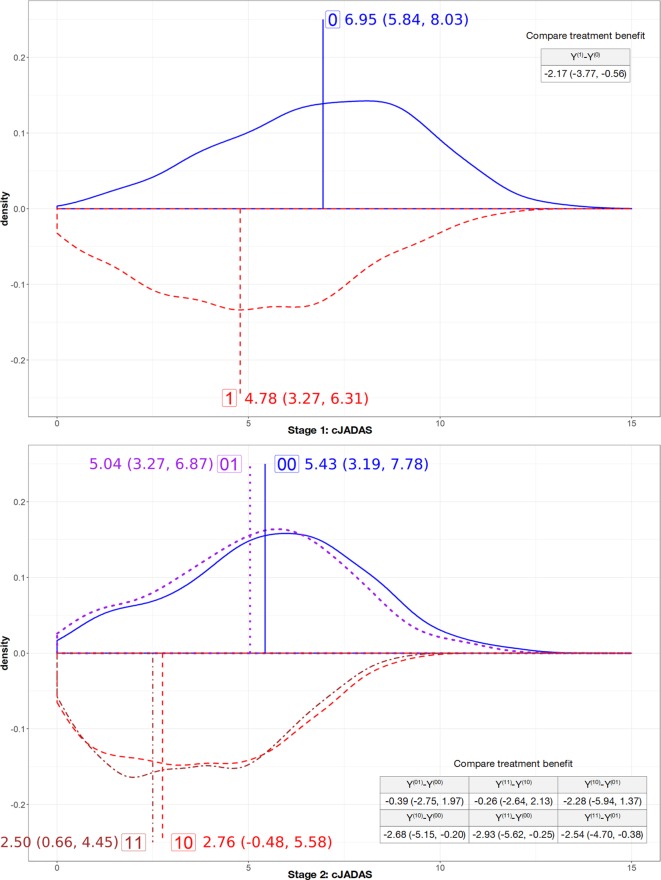
Density plots for estimated potential clinical Juvenile Arthritis Disease Activity Score (cJADAS) outcome. Average treatment effect and compared treatment benefits are reported in mean and 95% CI.

After balancing out the treatment selection bias (figure 4), causal inference analyses predicted 6-month and 12-month cJADAS for all patients had they gone through different treatment strategies. The treatment benefit, contrasting different treatment strategies, were estimated and presented also in figure 5. At 6 months of follow-up visit, the estimated mean cJADAS was 4.78 (95% CI 3.27 to 6.31) and corresponds to the reduction (∆) from the baseline cJADAS by a mean±SD of 8.87±0.80 if all were treated on cs+bDMARD versus 6.95 (95% CI 5.84 to 8.03; ∆=6.70±0.58) if they were treated on the csDMARD. The early aggressive treatment, on average, expected to reduce cJADAS by additional −2.17 (95% CI −3.77 to −0.56) at 6 months.

After 6 months of treatment, the initial treatment was adjusted by prescribing on or withdraw bDMARDs from the initial prescription. Following the initial csDMARD treatment, the expected mean cJADAS was 5.04 (Y^(01)^, 95% CI 3.27 to 6.87; ∆=8.61±0.92) if introducing bDMARDs versus 5.43 (Y^(00)^, 95% CI 3.19 to 7.78; ∆=8.22±1.18) if continuing on csDMARDs. The average treatment benefit was only −0.39 (Y^(01)^-Y^(00)^, 95% CI −2.75 to 1.97). Similarly, adjustment made following the initial cs+bDMARD led to nearly identical cJADAS outcome with the estimated mean cJADAS being 2.50 (Y^(11)^=continue on bDMARD, 95% CI 0.66 to 4.45; ∆=11.15±0.97) and 2.76 (Y^(10)^=withdraw bDMARD, 95% CI −0.48 to 5.58; ∆=10.89±1.53). Additional analyses suggested that the odds for the early use of bDMARD (Y^(10)^) to be more effective than the later use (Y^(01)^) is 8.25, and the odds to be at least one point more effective in cJADAS is 3.15. The analyses did not identify heterogeneous treatment effect by JIA subtypes or baseline cJADAS. Sensitivity analyses reported consistent results (presented in online supplementary material).

10.1136/rmdopen-2019-001091.supp2 Supplementary data

### Secondary outcome: PedsQL generic total score at 12 months

Similarly to the cJADAS results, the early aggressive group presented worse quality of life at the baseline, but similar scores at the 6 and 12 months of follow-up, with PedsQL scores (mean±SD) 61.35±19.24 versus 67.58±17.53 at baseline, 73.40±17.42 versus 74.34±19.38 at 6 months and 76.32±16.47 versus 78.52±18.63 at 12 months. The box–whisker plots of PedsQL score that over time were grouped by initial prescription are presented in figure 3B.

Patients were asked to complete the PedsQL generic module on an annual basis, thus the causal inference analyses could only evaluate the effectiveness for the 12-month outcome. The results reported estimated PedsQL scores of 76.26±4.80 and 82.61±6.09 after 12 months treated on the csDMARDs and cs+bDMARDs, respectively, showing 6.35 (95% CI −5.89 to 18.58) points improvement for cs+bDMARDs versus csDMARDs. Both presented clinically meaningful improvement from the baseline, ∆=15.17±6.10 and ∆=8.82±4.81 improvement in cs+bDMARDs and csDMARDs, respectively.

## Discussion

A window of opportunity may exist where early effective DMARD treatment could address underlying disease pathophysiology, prevent structural damage in joints and prevent functional impairment.^4 8 21–23^ This study offers real-world evidence supporting the effectiveness of early aggressive treatment, consistently with the results from existing RCTs. A multicentre randomised open-label clinical trial suggested that the combination of infliximab and methotrexate was better in achieving clinically inactive disease or minimal disease activities than csDMARD alone.^13^ The double-blinded Trial of Early Aggressive Therapy in Polyarticular Juvenile Idiopathic Arthritis (TREAT) study found that the combination treatment of etanercept and methotrexate+prednisolone achieved more clinical remission on medication than methotrexate+prednisolone.^21^ Both trials were not new patient DMARD-naive designs nor compared different adaptive treatment strategies. Despite the differences, our study reached a similar conclusion, confirming better clinical effectiveness of the early aggressive treatment strategy.

Little is known about the effectiveness of early aggressive approach on quality-of-life outcomes. Although not statistically significantly different, the 6.35 (95% CI −5.89 to 18.58) treatment effect in PedsQL scores at 12 months was greater than the established minimal clinically important difference of 4.5.^16^ The ongoing prospective cohort study evaluating the effectiveness of consensus treatment plans for pcJIA is expected to bring more clarity.^24^

To the best of our knowledge, this is the first study that provides real-world evidence of effective early initiation of bDMARD treatment. In the routine clinical care, treatment often is adjusted based on patients’ disease progress. Within an established EMR system, such interactions could be tracked from the first date of diagnosis throughout the course of disease progression and treatment, particularly for patients with chronic conditions. Therefore, it is an invaluable data source for evaluating the effectiveness of different timing of treatment initiation or treatment withdraw, as well as understanding potential treatment heterogeneity. This study demonstrates, with careful data management and data quality assurance, that the EMR could be used for better understanding of treatment effectiveness. Detailed steps in ensuring data quality from EMR have been reported elsewhere.^25^

### Limitations and generalisability

As an observational comparative effectiveness research (CER) using EMR data, this study is limited in several ways. First, the treatments were determined by medication prescription recorded in EMRs. Records of actual medication dispensing and treatment adherence were not available in EMRs. Second, patients in routine clinical care did not necessarily follow the predetermined schedule of follow-up and made it challenging to evaluate the observational CER at given time points. The existence of unmeasured confounders could bias the causal inference analyses results. This study conducted sensitivity analyses by further adjusting the quality-of-life measures. The sensitivity analyses results found that inclusion of additional PedsQL measures in these causal inference analyses resulted in nearly identical results as the primary analyses. Further, the study did not investigate safety outcomes because the data could not be reliably extracted retrospectively using EMR data. Lastly, a common approach adopted in clinic is to decide after 3 months on methotrexate if a bDMARD should be introduced. Future study should consider investigating three-staged adaptive treatment (3, 6 and 12 months).

The generalisability of this study is limited due to single-centre data. Patients from different centres may represent a different patient population in their demographics and disease subtypes. Clinicians from different centres may also engage different practices in treatment assignment. Our study did not find significant subgroup treatment effect. However, the study could be limited by the small sample size particularly for the less prevalent subtypes. Physician global assessment and patient/parent global assessment of well-being could be subject to individual and centre variations; thus, the effect size may differ by clinical centres. Future studies should consider using multicentre data and investigate further on subgroup treatment effect.

At last, the study only examined the cJADAS and PedsQL up to 12 months. It did not consider adverse events nor long-term outcomes. Inactive disease is the ultimate goal of the treatment, and future studies should compare the alternative treatment strategies in achieving inactive disease and in longer term of follow-up.

Despite these limitations, the study included patient care data obtained from patients during their routine clinical care. Thus, the study offers real-world evidence that is generalisable to the routine clinical practice in treating patients with pcJIA.

## Conclusions

All treatment strategies show significant improvement from the baseline. The study results suggest that, compared with csDMARD only, early aggressive use of bDMARD in treating patients with pcJIA soon after diagnosis achieves more than two points of additional reduction in disease activity at 6 months. Adding bDMARD after 6 months to the initial treatment provides very little added benefit. Future studies are needed to investigate the long-term clinical and health-related quality-of-life outcomes.
