# Supplementary material for: Timing matters: real-world effectiveness of early combination of biologic and conventional synthetic disease-modifying antirheumatic drugs for treating newly diagnosed polyarticular course juvenile idiopathic arthritis
Source: RMD Open. 2020 Feb 2;6(1):e001091. doi: 10.1136/rmdopen-2019-001091 (PMC7003379; doi:10.1136/rmdopen-2019-001091)
Supplement: Supplementary data [file rmdopen-2019-001091supp001.pdf]

Technical Supplement: GPMatch Causal Inference Method

1. Introduction

In routine clinical practice, treatments are assigned deliberately by patients' disease status. Sicker patients tend to receive more aggressive treatment. Therefore, the correlation between the treatment and the outcomes using the data collected from the real world cannot be used to inform treatment effect directly. Causal inference methods are specifically designed to remove the treatment-by-indication bias inherent in observational data for understanding the comparative effectiveness of treatment effect much like what we would obtain from conducting a randomized controlled trial.

Most of the causal inference methods are developed considering single point treatment assignment that treatment is assigned only once and stays the same over time. In treating patients with chronic illness or prolonged condition, however, treatments are often time-varying, adaptive according to the patient's progress and responses to the previous treatment assignment. In our study, as depicted in Figure 1, the DMARD treatment is adaptive – the stage-2 treatment assignment was determined based on how well patients progress after the initial treatment assignment. In other words, the patient disease activities observed at the 6 months is the key consideration for the next medication prescription. In addition, the 6 months disease activities are major determinants to the 12 months study outcomes. Therefore, the 6 months disease activity measures are time-dependent confounders and also immediate outcomes at the 6 months. Simply controlling for these intermediate disease activity measures will control away the true treatment effect of the time-varying adaptive strategy (Daniel et al. 2013; J. M. Robins, Hernán, and Brumback 2000). Therefore, linear mixed modeling that simply includes the covariates cannot be used. The commonly used causal inference methods designed for the point treatment assignment will not appropriately account for the time-dependent confounders, and thus they cannot be used directly.

Daniel et al. (2013) provided a comprehensive review of three existing causal inference methods for dealing with time-dependent confounding, where g-computation formula is one of the most commonly used method. The g-computation formula was first suggested by Robins (J. Robins 1986). The idea is to estimate the expected potential outcomes one stage at a time, then compute the missing potential outcomes at each stage, and then compute the missing potential outcomes. The method can be implemented using the many regression modeling techniques. When correctly specified, it provides accurate estimate of treatment effect. Recently, Keil et al. (2018) presented a parametric Bayesian's g-computation formula and suggested it can improve the accuracy of estimates of causal effects in small samples or sparse data. The parametric modeling of causal inference could suffer when model is mis specified.

Flexible parameter rich Bayesian models can lessen the concern over model mis specification. For example, Roy et al. (Roy, Lum, and Daniels 2017) presented a nonparametric Bayesian's marginal structural model utilizing both Dirichlet and Gaussian Process priors for point treatment assignment.

The GPMatch is a Bayesian nonparametric g-computation formula method. It utilizes the GP prior to offer a nonparametric flexible regression model, it also formulates GP covariance function as a matching tool, such that it resembles matching to control for the confounding bias. It can predict the missing potential outcomes by a weighted sum of observed data, with larger weights assigned to those data points in closer proximity and smaller weights to those data points further away. This appendix provides technical details of the method.

2. Point Treatment Assignment

For the ease of presentation, we first present the GPMatch method by considering a point treatment assignment ($\mathrm{A_i}$). The GPMatch model fits the observed outcome data Y_i by a regression model on the sample covariates (X_i),

$$Y_i = X_i\beta + \mathrm{A_i}\tau + \eta_\mathrm{i} + \varepsilon_i, \qquad (1)$$

for $i = 1, \dots, n$, where $\eta \sim GP(0, .)$, and $\varepsilon_i \; iid \sim N(0, \sigma_0^2)$.

Specifically, we define the GP Covariance function being proportional (denoted by ∝ symbol) to the squared exponential of the distance d_{ij},

$$Cov(Y_i, Y_j) \propto exp(-d_{ij}^2),$$

Where d_{ij}=distance in the baseline covariate space between the ith and the j^{th}patients, for $i = 1 \dots n,\; j = 1, .. n.$ In other words, for any pair of patients, we assume their correlations are proportional to the distance (d_{ij}) between the two. The value of d_{ij} is determined by the pre-treatment covariates of each pair of patients. For example, we may define

$$d_{ij} = \sqrt{\sum_{k=1}^{q} \frac{|v_{ki} - v_{kj}|^2}{\phi_k}},$$

where v_{ki} and v_{kj} are values of the pre-treatment covariates, for $k = 1, \dots, K.$ Of note, the V variable could be a subset of X, centered to mean 0 and scaled to variance of 1. For example, the v_{ki} could be standarized values of MD global, active joint count and patient wellbeing for a given patient prior to treatment assignment; while X could include the post-treatment variables such as the follow up time. Following such GP prior specification, patients with similar disease activities are considered highly correlated. On the other hand, patients with very different presentations of disease activity are only weakly or not correlated. The parameter ϕ_k

determines the distance beyond which patients are considered independent. It is estimated from fitting the data.

After fitting the above GP regression, GPMatch estimates the missing potential outcomes for any given patients (denoted by subscript *) by

$$\hat{Y}_*^{(a)} = a\hat{\tau} + \sum_{i=1}^{n} \widehat{w}_{*i}(Y_i - A_i\hat{\tau}),$$

Where $\widehat{w}_{*i}$ is the i-th element of the standardized $\boldsymbol{w}_*$ such that $\sum_{i=1}^{n} \widehat{w}_{*i} = 1$, with $\boldsymbol{w}_* = \boldsymbol{k}_*(v)' \boldsymbol{\Sigma}^{-1}$, $\boldsymbol{k}_*(v)_{n\times 1} = \left\{\widehat{Cov}(Y_*, Y_i)\right\}_{i=1}^{n}$, $\boldsymbol{\Sigma}_{\mathrm{n\times n}} = \left\{\widehat{Cov}(Y_i, Y_j)\right\}_{i,j=1}^{n}$. We see $\widehat{w}_{*i}$ is proportion to the estimated covariance between the given patient and each i-th patients in the observed data. The $\hat{\tau}$ is the estimated treatment effect. With the squared exponential distance covariance function defined above, the w_i quickly decline as the distance increase. Thus, the estimate of $\hat{Y}_*^{(a)}$ is primarily determined by a limited set of data points from patients who are part of the “matching” neighborhood. For this reason, we name this method GPMatch.

The squared exponential distance function can be considered as an alternative distance measure to the Mahalanobis distance (MD), which is defined by

$$MD_{ij} = \sqrt{\left(v_i - v_j\right)' S^{-1} \left(v_i - v_j\right)} \quad if\ |v_{ik} - v_{jk}| < c, for\ k = 1,2, \dots, q; \qquad \infty \quad otherwise,$$

where $c \in R^+$ is the chosen caliper for MD matching, *S* is the sample variance-covariance matrix of confounding variables *V*. The MD matching requires specification of a caliper. Smaller c leads to more precise matching but often results in a serious reduction in sample size after matching. In GPMatch, no caliper is required. Instead, the length scale parameters (ϕ_k), which governs the extent to which the data points are matched, are estimated from the data. The GPMatch allows different length scale parameters for different confounding variables, such that it acknowledges that some confounders may play a relatively more important role in matching than other confounders. The variables with larger value of ϕ_k parameters are considered more important than those with smaller values.

For checking balance, we compare the two treatment groups on a central tendency and a dispersion measure for each of the k-th baseline pre-treatment covariates, $k = 1,..,q$. Both central tendency and dispersion can be expressed as a general function $g_k(a)$, for $a = 0,1$:

$$g_k(a) = \frac{1}{n_a} \sum_{\substack{i=1 \\ A_i=a}}^{n} g\left(X_{ki} - \tilde{X}_{ki}\right),$$

where, $\tilde{X}_i = \sum_{j=1}^{n} \hat{w}_{ij} X_j$, a weighted sum of observed baseline covariates. The weights are estimated from fitting GPMatch model. For central tendency measure, $g_k(.)$ is the identical function; for the dispersion measure, $g_k(.)$ is the absolute value function.

To assess the extent to which GPMatch is able to achieve better balance, we compare the balance measure against the unadjusted simple linear regression model $Y \sim A$, in which case $w_{ij} = 1$ and $\tilde{X}_{ki} = \bar{X}_k$. Thus under the identical function, we have a group mean difference

$$g_k(1) - g_k(0) = \bar{X}_{k1} - \bar{X}_{k0}.$$

Under the absolute value function, we have a group difference on the mean absolute deviation (MAD)

$$g_k(1) - g_k(0) = \sum_{i \in (A_i=1)} |X_{ki} - \bar{X}_k| - \sum_{i \in (A_i=0)} |X_{ki} - \bar{X}_k|.$$

In a randomized trial setting, it is easy to see that both measures are expected to be 0.

In the GPMatch, $\tilde{X}_i = \sum_{j=1}^{n} \hat{w}_{ij} X_j$ is estimated by the weighted average data points from the "matched" neighborhood. When matched well, the method should be able to achieve comparable location and dispersion measures between two treatment groups. Plotting these two measures from GPMatch (after adjustment) compared against the same measures from the simple linear regression (before adjustment) allows us to visually inspect the balance after GPMatch. It also offers a way of selecting baseline covariates that needs to be balanced.

3. Time-varying Adaptive Treatment

Let $Y_1^{(a_0)}$ denote the potential outcome 6-month cJADAS score that would have observed had the patient treated on the bDMARD ($a_0 = 1$) or nbDMARD ($a_0 = 0$) at the time of diagnosis. Let $Y_1^{(a_0,a_1)}$ denote the potential outcome cJADAS score at the 12-month that would have been observed had the patient treated by the a_0 at the baseline followed by a_1 at the 6-month, where a_t corresponds to the nbDMARD($a_t = 0$) and bDMARD($a_t = 1$) treatment at the time $t = 0\ (baseline), 1(12 - month)$. X_0 denote the baseline covariates, and $X_1(a_0)$ denote the covariates measures, including disease progression, at the 6-month pre- 2nd-stage treatment assignment after treated by a_0. To answer the study CER questions, we focused on estimating the following average causal treatment effect.

1) The average treatment effect at stage 1 (ATE@stage1): $\hat{E}\left(Y_1^{(1)} - Y_1^{(0)} \middle| X_0\right)$; This is the treatment of the initial DMARD assignment.
2) The average treatment effect at stage 2 conditional on the past treatment assignment and the patient's progression at the 6-month (CATE@Stage2): $\hat{E}\left(Y_2^{(a_0,1)} -\right.$

$Y_2^{(a_0,0)} \Big| X_0, X_1(a_0) = x_1, Y_1(a_0) = y_1)$. This is the treatment effect of the 2nd stage DMARD assignment given the past treatment and disease progression.

3) The marginal average treatment effect at the 12-month (the study endpoint) (MATE@Stage2): $E\big(Y^{(a_0,a_1)} - Y^{(a_0',a_1')} \big| X_0\big)$. This is the overall treatment effect over the 12 months, following different treatment sequences. Of note, this treatment effect is averaged over the intermediate responses to the initial treatment response.

For evaluating causal treatment effects of time-varying adaptive treatments, the GPMatch for the point-treatment assignment can be easily extended following the Bayesian's g-computation formula. The GPMatch model predicts the posterior of the missing potential outcomes at each decision point, in a sequential generative manner. The potential outcomes for any given treatment history are estimated, and the averaged treatment effect is estimated by the contrast between an intervention vs. an comparator adaptive treatment strategies (ATS) at the final study endpoint. Finally, optimal ATS can be identified by maximizing the potential outcomes. Box 1 outlines the algorithm used for a two-stage ATS assignment.

Box 1. GPMatch Algorithm for 2-stage Adaptive Treatment Strategy Assignment
1. Stage-1 Modeling<br>1.1. Fit the GPMatch model for all the observed intermediate outcomes X_{i1} immediately prior to the second treatment decision point, by weighted matching on important baseline covariates ($X_{i,0}$). Here X_{i1} includes the outcome of interest (e.g. cJADAS scoer at the 6 month) and other disease progression measurements (e.g. AJC, LOM, ESR measures at the 6 month) to assess how well patients responded to the first treatment assignment.<br>1.2. Check balance on the mean and the MAD between two groups on all their baseline pre-treatment assignment variables. Including any additional covariates if the balance has not been achieved.<br>1.3. Generate posterior MCMC for all model parameters, estimate posterior of $[\hat{X}_{i,1}(0), \hat{X}_{i,1}(1) \mid A_{i,0}, X_{i,0}, X_{i,1}]$ for each patient using the Stage-1 model. Save the predicted $\hat{X}_{i,1}(0), \hat{X}_{i,1}(1)$ for the later g-computation step.<br>1.4. Estimate the ATE@stage1 for intermediate outcome and for all intermediate treatment response covariate measures.<br><br>2. Stage-2 Modeling<br>2.1. Fit GPMatch model for the final outcome Y_i. Here, Y_i is the cJADAS score at the 12-month. The GPMatch matches patients on their baseline treatment (A_0), baseline covariates (X_{i0}) and the treatment responses (X_{i1}) measured at the end of first stage. This is because the second stage treatment assignment (A_1) is determined adaptively in response to the patients' first stage assignment, patients' initial disease status and responses to the initial treatment assignment.<br>2.2. Check balance on all 2-stage pre-treatment covariates and ensure all stage-2 covariates are balanced.

2.3. The GPMatch estimates the treatment effect from the second stage.
2.4. Generate posterior MCMC for all second stage model parameters, and estimate the posterior of $[\hat{Y}_i(00), \hat{Y}_i(01), \hat{Y}_i(10), \hat{Y}_i(11) | A_{i,1}, A_{i,0}, Y_i, X_{i,1}, X_{i,0}]$
2.5. Estimate the conditional ATE, CATE@stage2 for treatment outcome at the end of second stage, conditional on the treatment history and patient responses at the end of stage1.

3. G-computation
3.1. Integrate out the intermediate responses, estimate the marginal posterior $[\hat{Y}_i(00), \hat{Y}_i(01), \hat{Y}_i(10), \hat{Y}_i(11) |\ X_{i,0}]$
3.2. Estimate the marginal ATE, MATE@stage2 for all patients

4. Software and Data Sharing

The GPMatch method has been implemented in a graphic user inference online application that is accessible to general public at https://pcats.research.cchmc.org. It offers capacity to study comparative effectiveness of both single point and 2-stage adaptive treatment strategies.

Data used in this CER study can be made available upon signing Data Sharing Agreement between two institutes.

Reference

Daniel RM, Cousens SN, De Stavola BL, Kenward MG, Sterne JAC. Methods for dealing with time-dependent confounding. *Stat Med*. 2013;32(9):1584-1618. doi:10.1002/sim.5686

Keil AP, Daza EJ, Engel SM, Buckley JP, Edwards JK. A Bayesian approach to the g-formula. *Stat Methods Med Res*. 2018;27(10):3183-3204. doi:10.1177/0962280217694665

Robins, James. 1986. “A New Approach to Causal Inference in Mortality Studies with a Sustained Exposure Period—Application to Control of the Healthy Worker Survivor Effect.” *Mathematical Modelling* 7(9–12): 1393–1512.

Robins, James M., Miguel Ángel Hernán, and Babette Brumback. 2000. “Marginal Structural Models and Causal Inference in Epidemiology.” *Epidemiology* 11(5): 550–60.

Roy, Jason, Kirsten J Lum, and Michael J Daniels. 2017. “A Bayesian Nonparametric Approach to Marginal Structural Models for Point Treatments and a Continuous or Survival Outcome.” *Biostatistics (Oxford, England)* 18(1): 32–47.
