# Supplementary material for: Timing matters: real-world effectiveness of early combination of biologic and conventional synthetic disease-modifying antirheumatic drugs for treating newly diagnosed polyarticular course juvenile idiopathic arthritis
Source: RMD Open. 2020 Feb 2;6(1):e001091. doi: 10.1136/rmdopen-2019-001091 (PMC7003379; doi:10.1136/rmdopen-2019-001091)
Supplement: Supplementary data [file rmdopen-2019-001091supp002.pdf]

Supplementary material

## S1: Sensitivity Analysis with PedsQoL Baseline Measures

The primary analyses did not include the PedsQL measure at the baseline. This sensitive analyses used the same method as described in the primary analyses, with including PedsQoL baseline measures (both generic and rheumatology modules) as additional confounders in the analyses in addition to those considered in the primary analyses.

The estimated cJADAS10 are presented in Figure S1. The result estimated mean±SD of 6.92±0.58 and 4.75±0.80 cJADAS score by 6 months if treated on the conservative treatment and early aggressive treatment respectively. Early aggressive treatment, on average, produced a significant -2.17 points (95%CI -3.78 to -0.56) reduction in the cJADAS compare with the conservative treatment at 6 months, same treatment benefit was sustained up to 12 months. The early aggressive treatment had the highest level of improvement (Δ=11.18±0.96) in cJADAS from baseline. Overall, the sensitivity analysis supported the same conclusions as the primary analyses.

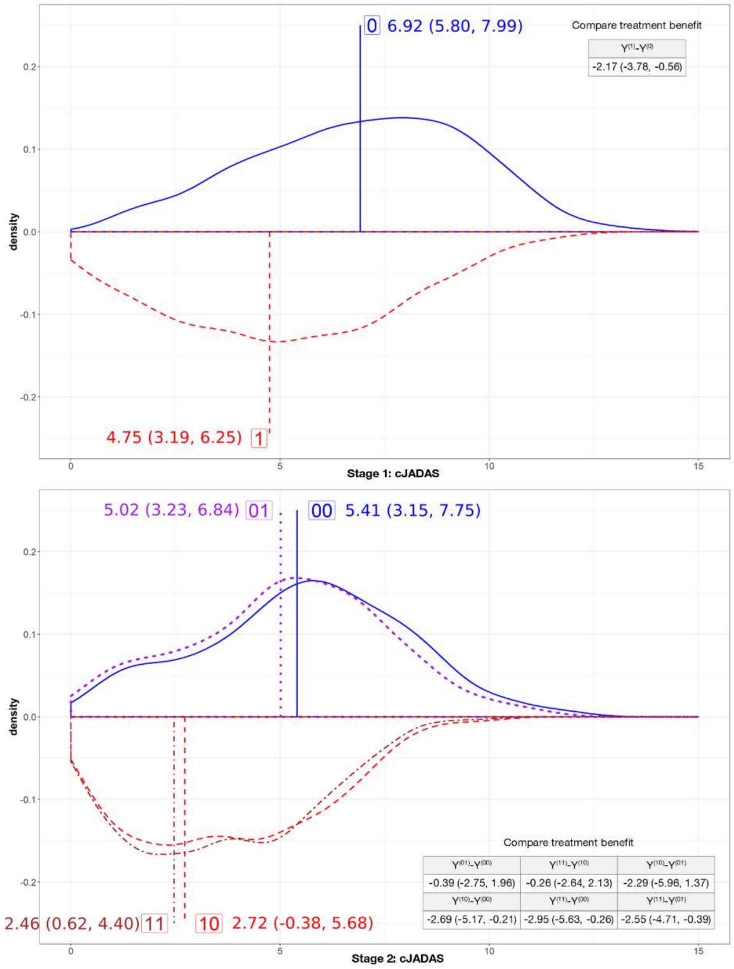


*Figure S1. Density plot of estimated cJADAS10 outcome using GPMatch method*
